# Wet chemistry route for the decoration of carbon nanotubes with iron oxide nanoparticles for gas sensing

**DOI:** 10.3762/bjnano.10.10

**Published:** 2019-01-09

**Authors:** Hussam M Elnabawy, Juan Casanova-Chafer, Badawi Anis, Mostafa Fedawy, Mattia Scardamaglia, Carla Bittencourt, Ahmed S G Khalil, Eduard Llobet, Xavier Vilanova

**Affiliations:** 1 Electronics & Communications Department, Faculty of Engineering, Arab Academy for Science and Technology & Maritime Transport, Cairo, Egypt; 2 MINOS-EMaS, Universitat Rovira i Virgili, Avda. Països Catalans, 26, 43007 Tarragona, Spain; 3 Spectroscopy Department, Physics Division, National Research Centre, 33 El Bohouth st. (former El Tahrir st.), P.O. 12622 Dokki, Giza, Egypt; 4 Chemistry of Interaction Plasma Surface (ChIPS), University of Mons, 7000 Mons, Belgium; 5 Physics Department & Center for Environmental and Smart Technology (CEST), Faculty of Science, Fayoum University, Fayoum, Egypt

**Keywords:** benzene detection, doping, gas sensor, metal nanoparticle decoration, multiwalled carbon nanotubes, NO_2_ detection, room temperature gas sensing, surface modification

## Abstract

In this work, we investigated the parameters for decorating multiwalled carbon nanotubes with iron oxide nanoparticles using a new, inexpensive approach based on wet chemistry. The effect of process parameters such as the solvent used, the amount of iron salt or the calcination time on the morphology, decoration density and nanocluster size were studied. With the proposed approach, the decoration density can be adjusted by selecting the appropriate ratio of carbon nanotubes/iron salt, while nanoparticle size can be modulated by controlling the calcination period. Pristine and iron-decorated carbon nanotubes were deposited on silicon substrates to investigate their gas sensing properties. It was found that loading with iron oxide nanoparticles substantially ameliorated the response towards nitrogen dioxide.

## Introduction

Carbon nanotubes (CNTs) are considered to be a very interesting material, especially after being rediscovered by Sumio Iijima in 1991 when he found multiwalled CNTs in carbon soot prepared by arc discharge [1]. During the past years, CNTs have proved to possess extraordinary electrical, mechanical, physical and chemical properties [2–3]. In particular, they have been extensively researched in gas sensing applications because of their high thermal and chemical stability, high adsorption capacity and suitability for being functionalized, which enables tailoring (to some extent) their sensitivity and selectivity to the chemical environment [2–5]. CNT gas sensors often exhibit fair sensitivity to gases even when operated at room temperature. Since their electrical conductivity is affected upon the adsorption of gases, their response is often measured as a change in resistance of a CNT film. The fact that CNT gas sensors can be intrinsically low-power devices make them very attractive for their integration in ubiquitous, unattended mobile sensing nodes running on small batteries or on energy harvested from their environment [4].

Among the wide range of functionalization strategies that can be envisaged for tailoring the selectivity of CNTs towards target gases, one of the simplest consists of decorating the outer wall of CNTs with metal or metal oxide nanoparticles [6–9]. In some cases, metal or metal oxide nanoparticles show interesting catalytic properties for the decomposition of target molecules into more reactive species that, in turn, interact with CNTs. In addition, such nanoparticles shift the Fermi level of CNTs, adsorb target molecules, and help in mediating the charge transfer between adsorbates and CNTs [6,10].

Several metal oxides have been reported as useful for decorating CNTs and improving their interaction with gas molecules. Sensitivity and selectivity can be tailored by selecting the type of metal oxide employed, the size of nanoparticles and the decoration density or loading [6,10–11]. Metal oxides have been extensively investigated for sensing a wide range of gases [12–14]. Among them, iron oxide is a semiconductor that has been used in many gas sensing applications because of its low cost and simple preparation [14–15]. This oxide has been used in the detection of acetone, H_2_S, several alcohols, CO, acetic acid and liquefied petroleum gas (LPG) [16] and forming composites with other materials such as graphene oxide or polyaniline has been reported to detect NO_2_ [17–18]. The decoration of CNTs with iron oxide has been reported for sensing different species in air such as acetone, CO_2_ and some volatile organic compounds [19–21]. Moreover, composites made of CNTs and iron oxide have been also used for sensing ammonia and NO*_x_* [22–23]. Among those gases NO_2_ is considered one of the most dangerous air pollutants occurring both indoors, due to using of gas stoves, and outdoors from fuel powered motor vehicles and power plants especially in long-term exposure conditions. As research studies show, exposure to this gas can lead to an increase in oxidative stress in the body, resulting in behavioral and learning-memory impairments. Also, there is a consistent relationship between NO_2_ and respiratory and asthmatic problems at mean daily concentrations (20–80 ppb) well below air quality guidelines [24–25], which indicates the importance of fabricating such a gas sensor to be used in different applications.

In this paper, we report on a wet chemistry route that was successfully employed to chemically modify CNTs by decorating them with iron oxide nanoparticles. This inexpensive method allows control of the decoration density and nanoparticle size. The effects of changing the process parameters on the morphology of CNTs, the size of iron oxide nanoparticles and the decoration homogeneity achieved are studied and discussed in detail. The morphology, quality and chemical composition of the iron oxide decorated carbon nanotube samples were investigated employing transmission electron microscopy (TEM), Raman spectroscopy and X-ray photoelectron spectroscopy (XPS).

The differently decorated CNT samples were used to make gas sensors for detecting nitrogen dioxide. A study of the gas sensing properties of the different hybrid nanomaterials was conducted in an effort to determine the optimal functionalization parameters to maximize sensor response. The selectivity of the resulting layer for potential interfering gases such as CO and benzene has also been investigated as well as the effect of ambient humidity.

## Experimental

### Materials

All materials and reagents used (listed below) were of analytical grade and were used as received.

- COOH functionalized multiwalled carbon nanotube (MWCNTs), Nanocyl (C purity higher than 95%)
- Nitric acid, Scharlau (HNO_3_ 68–70%)
- Sulfuric acid, J. T. Baker (H_2_SO_4_ 95–97%)
- Conductive silver paste, Sigma-Aldrich
- Methanol, Scharlau (CH_3_OH 99.9%)
- Ethanol, Scharlau (C_2_H_5_OH 96% extra pure and 99.5% absolute)
- Acetone, Scharlau (C_3_H_6_O 99.5%)
- Dimethylformamide (DMF), Alfa Aesar (C_3_H_7_NO 99.8%)
- Iron(III) nitrate nonahydrate, Sigma-Aldrich (Fe(NO_3_)_3_·9H_2_O 99.95% trace metal basic)
- Acetic acid, Fluka Analytical (CH_3_COOH 99.8%)

### Decoration and characterization of carbon nanotubes

Commercial CNTs from Nanocyl functionalized with (COOH) groups were further chemically purified by an acidic treatment to remove any traces of catalyst or amorphous carbon. This treatment also helps in creating more active sites (e.g., some defects) on the side walls of the carbon nanotubes, preparing them for the decoration process. A mixture of H_2_SO_4_ and HNO_3_ was prepared at a ratio of 3:1. 200 mg of CNTs were mixed with 12 mL of the acidic mixture and were stirred for one hour at room temperature. The reaction was exothermic and no cooling or water baths were used. During the reaction, ultrasonication was employed for the first 15 minutes only to assure the debundling of CNTs without damaging them. During the remaining 45 minutes, the mixture was stirred employing a magnetic stirrer [19,26–27].

When the acidic treatment was completed, the resulting black slurry was filtered out from the acidic mixture using vacuum filtration and then washed with DI water for several washing cycles until the pH was neutralized. Then, the neutral black slurry was dried in a drying oven at 80 °C for 4 hours.

For the decoration of carbon nanotubes, iron(III) nitrate nonahydrate was used as the iron precursor. 50 mg of the acidic-activated carbon nanotubes were added to 50 mL of solvent together with a corresponding amount of Fe(NO_3_)_3_·9H_2_O salt. Different solvents, methanol, ethanol, acetone and DMF, as well as different amounts of salt (with ratios 1:1, 1:1.3 and 1:1.5 for CNT/Fe weights) were tested to check the effect of both parameters in the effectiveness of the decoration. The mixtures were stirred using a magnetic stirrer for 60 minutes. In a first attempt, the mixtures were heated to 80 °C to completely evaporate the solvent. This approach did not succeed, as commented in the results and discussion section, so a new approach was designed. In this second attempt, the mixtures were heated to 80 °C until 40 mL of the solvent was evaporated. The remaining solution was then ultrasonicated for 15 minutes then heated at 80 °C on a hotplate with a magnetic stirrer till the complete evaporation of the solvent. Once dried, the resulting powder was exposed to vapors of acetic acid for 15 minutes and later heated for 20 minutes at 80 °C to remove all the physically absorbed acetic acid [28]. Finally, the powder was calcined at 450 °C during either 15 or 30 minutes. In this way the effect of the calcination time on the decoration process was also evaluated.

The chemical composition of the decorated CNTs were measured by X-ray photoelectron spectroscopy (XPS) using a Versaprobe PHI 5000 from Physical Electronics, equipped with a monochromatic Al Kα X-ray source at a base pressure of about 10^−9^ mbar. The sample powders were mounted on double-sided conductive vacuum tape. The X-ray photoelectron spectra were collected at a take-off angle of 45° with respect to the electron energy analyzer and the spot size was 200 µm. A pass energy (PE) of 20 eV was used for the high-resolution spectra (Fe 2p, C 1s and O 1s), while PE = 100 eV was used for the survey spectrum, accounting for an overall energy resolution of about 0.5 eV. Different points on each sample were measured in order to ensure the homogeneity. The chemical composition was then evaluated by using CASA XPS software.

TEM images were collected using a JEOL 1011 transmission electron microscope operating at 100 kV. The samples were dispersed in ethanol and a drop of resultant suspension was poured on carbon-coated copper grids.

The Raman spectra for the different samples was characterized using a Renishaw inVia spectrometer as the powder samples were mounted on clean glass slides. The samples were excited with a green (514 nm) laser using 50% laser power and the exposure time was 10 s.

X-ray diffraction (XRD) patterns were recorded at room temperature using a 202964 Panalytical Empryan diffractometer (Central Laboratory, Beni Suef University, Egypt) with a Cu Kα monochromatic radiation (*k* = 1.54056) operating at 40 kV and 30 mA from 5.0200° to 79.9800° with a 2θ step size of 0.0400 and a scan step time of 0.50 s in a continuous scanning mode.

### Fabrication and testing of gas sensors

In order to check the effect of the different decorations on the gas sensing properties of modified CNTs, simple sensing devices were fabricated. For that purpose, small rectangular pieces of a silicon wafer, previously oxidized at 1100 °C for 6 hours, were used as substrate. Heaters were attached to the back side of the sensor substrate using silver paste from Sigma-Aldrich. The substrates were heated in the oven at 120 °C for 30 minutes to cure the silver paste.

A dispersion in DMF was prepared with a concentration of 0.1 mg/mL of the modified CNT samples to be deposited. Then, the solution was ultrasonicated for 15 minutes before being deposited onto the silicon substrates. Two different approaches were used for depositing iron-loaded CNT films on the substrates: drop coating and air brushing. In drop coating, the substrate was heated on a hot plate up to 160 °C while the dispersion of CNTs in DMF was dropped by means of a pipette (drop by drop). The DMF was instantaneously evaporated when the drop was in contact with the heated silicon substrate, leaving the iron-loaded CNTs physically attached to it. In air brushing, the substrate was also heated to 160 °C while the suspension of CNTs in DMF was airbrushed onto the top of the substrate. As in the previous case, the solvent was instantaneously evaporated when in contact with the heated substrate, leaving the iron-loaded CNTs physically attached to it. To delimitate the area where the CNTs were deposited, a shadow mask of adhesive Kapton was used. This second approach leads to thinner active layers. The differences in the results for both approaches can be seen in the Supporting Information File 1 (Figure S1).

After the previous steps, heaters and the CNT layer were connected to a printed circuit board (PCB) using platinum wires. Those wires were attached to the heaters and CNT layers using silver paste that was cured in an oven at 120 °C for 30 minutes. To connect the Pt wires to the PCB we used tin wire and a soldering iron.

Wire bonded sensors ready for testing can be seen in the Supporting Information File 2 (Figure S2). The silicon substrates onto which carbon nanotubes were deposited were glued to an alumina substrate that included a platinum heater employing a thermally conductive epoxy.

A teflon chamber, which allowed allocating up to four sensors, was used for testing the gas sensing properties of the different nanomaterials. This chamber is shown in Supporting Information File 3 (Figure S3). A gas cylinder with a 100 ppm NO_2_ concentration balanced in dry air was used jointly with a set of mass flow controllers to generate the desired concentrations. The gas flux was fixed to 100 sccm during the whole experiments. The sensor response is defined as

[1] $$\left(R_{\text{G}}-R_{0}\right)/R_{0}$$

where *R*_G_ is the sensor resistance when exposed to NO_2_ and *R*_0_ is the sensor response when exposed to air.

Before starting any experiment, the sensors were heated while dry synthetic air was flowed in order to clean the sensor surface. Afterwards, different concentrations of NO_2_ gas (5 ppm, 10 ppm, 20 ppm, 50 ppm and 100 ppm) were successively pumped into the test chamber with the sensors operated at room temperature. The cycles consisted of 30 minutes of exposure to NO_2_ diluted in air and 3 hours of recovery in dry air.

To determine the selectivity of the sensor for carbon monoxide (CO) and benzene (C_6_H_6_), gas cylinders with a 100 ppm CO and 10 ppm C_6_H_6_ concentrations respectively, balanced in dry air, were used with the previously described set-up. To create the desired humidity in order to check the effect of moisture on the sensor response, a controlled amount of water was mixed with the gas flow by means a liquid mass flow controller.

## Results and Discussion

### Acidic purification and decoration of carbon nanotubes

**Figure 1:**
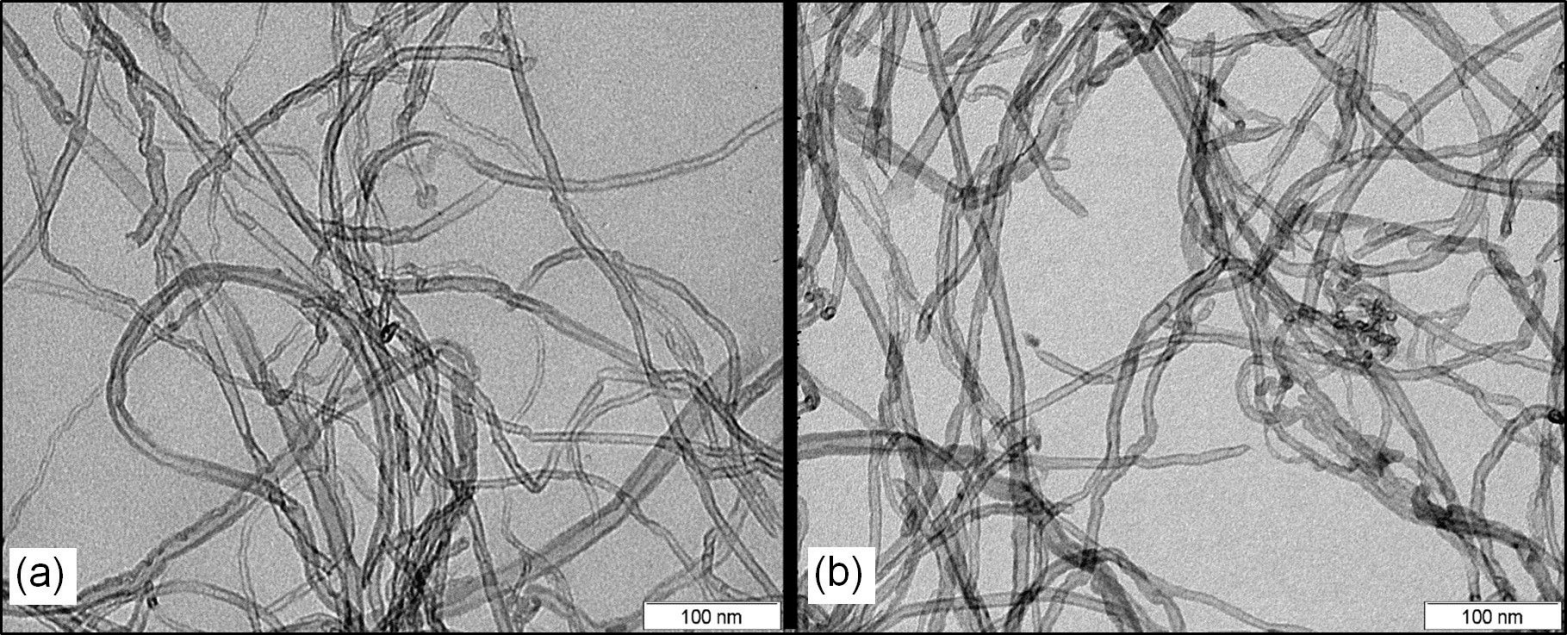
TEM images for COOH–CNTs (a) before acidic treatment and (b) after acidic treatment.

Figure 1 shows TEM images for the CNTs before and after the acidic treatment and, as shown, no significant changes are visible in the carbon nanotubes.

Since using the first approach for the decoration of CNTs led to the formation of large agglomerates of iron-loaded carbon nanotubes that could not be dispersed even after a sonication process in ethanol for 30 minutes (as can be seen in Figure S4, in Supporting Information File 4), this first approach was discarded and we focused our efforts in the second approach.

**Figure 2:**
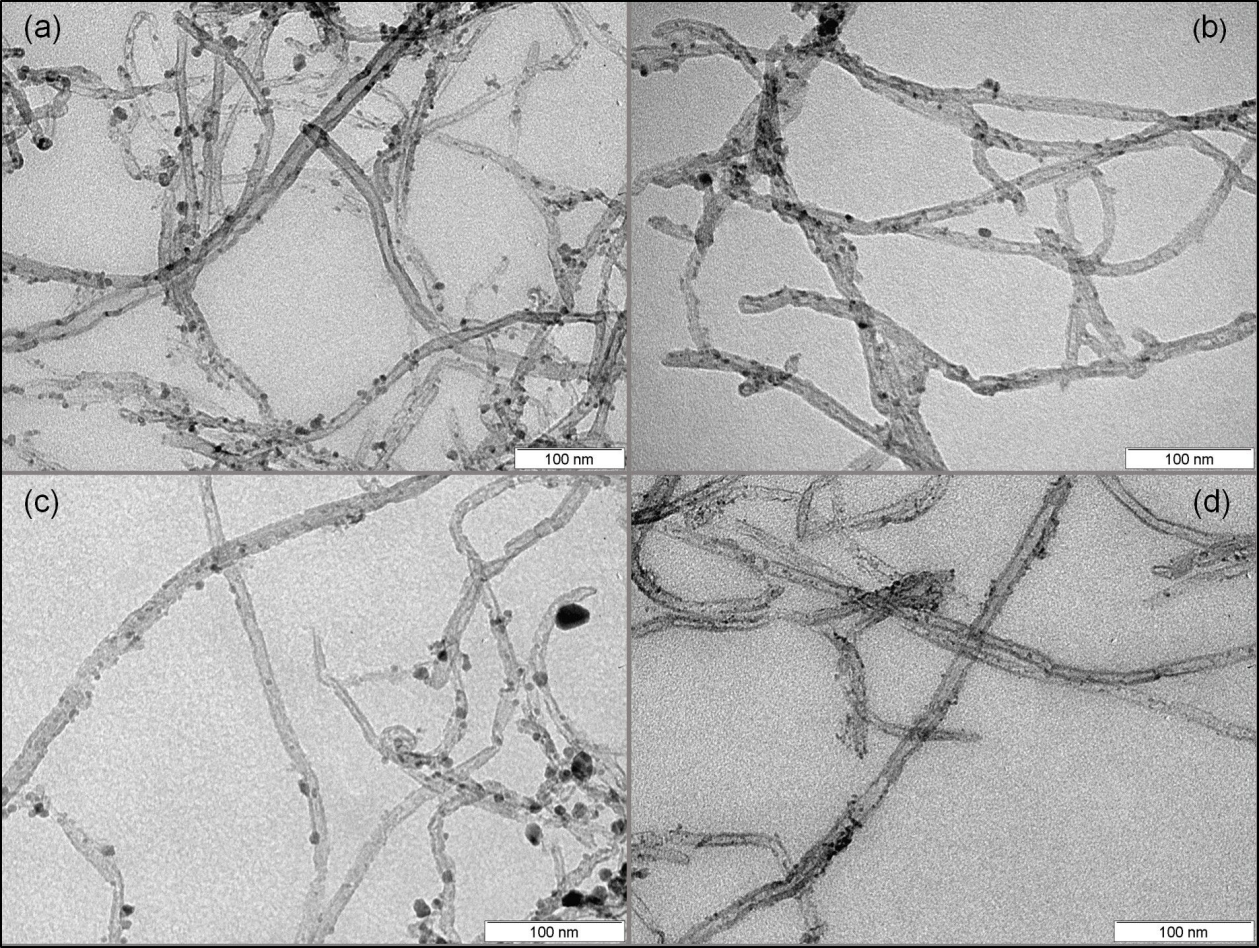
Different decoration homogeneity using different solvents, methanol (a), ethanol (b), DMF (c) and acetone (d).

Accordingly, the next step was to determine the best solvent for obtaining a well dispersed powder with homogeneous nanoparticle coverage. To analyze the effect of the solvent on the nanoparticle distribution, solutions with the four solvents considered (ethanol, methanol, acetone and DMF) were prepared using a 1:1.5 proportion in weight between carbon nanotubes and iron(III) nitrate nonahydrate and calcined for 30 minutes. TEM images of the results are summarized in Figure 2. As can be seen, both ethanol and methanol led to a homogenous distribution of the nanoparticles onto the carbon nanotubes. Moreover, the size of the nanoparticles in those cases was also homogeneous. Nevertheless, the decoration homogeneity was slightly better for methanol than for ethanol. On the other hand, acetone and DMF had a negative effect on both the decoration distribution and particle size. As we can see in the case of acetone, the dispersion of the nanoclusters is not uniform or homogenous and some areas have high decoration density while some other areas have very low decoration density. Also, in the case of DMF we can notice that dispersion of the nanoclusters was better than for acetone, but still some areas have a high density of decoration in which agglomerates of large particle size are formed.

According to these results, methanol was chosen as the most suitable solvent to be used in the production of iron-loaded CNT samples for further analysis, including the production of gas sensors.

**Figure 3:**
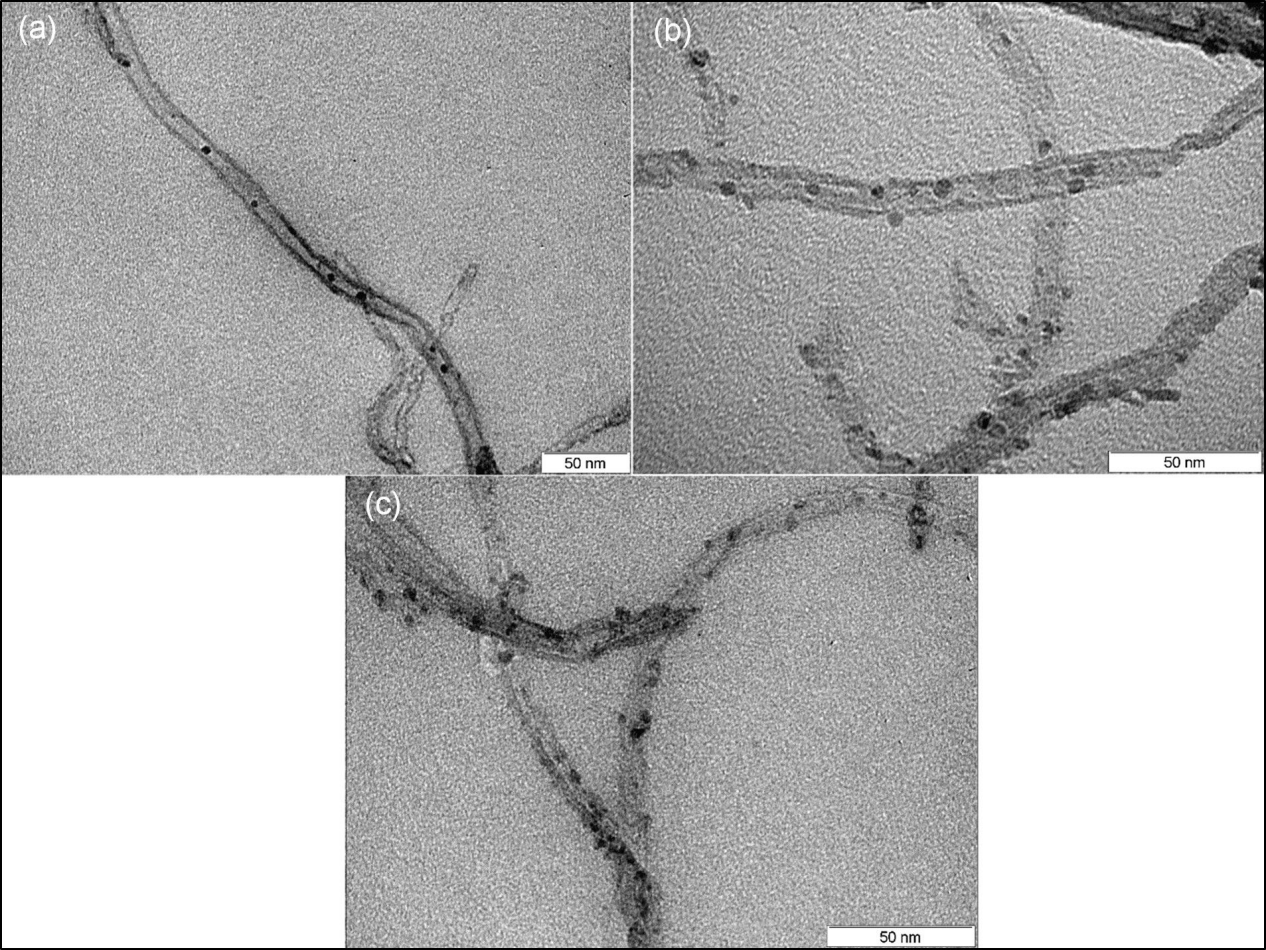
Different decoration densities for different decoration ratios of 1:1 (a), 1:1.3 (b) and 1:1.5 (c).

Once the best solvent was identified, it was necessary to determine the effect of the amount of iron oxide precursor employed on the decoration results (i.e., density and homogeneity of the loading, particle size). TEM images for different samples with different CNT/iron salt ratios were taken to investigate their effect on the decoration density, as shown in Figure 3. We can see that decoration density of CNT/Fe oxide increases by increasing the amount of iron salt, while the particle size was not affected.

Statistical analysis for the three samples shows that the average particle size of the NP does not increase when increasing the decoration dose. The mean particle size was found to be 3.44, 3.46 and 3.31 nm for decoration ratios of 1:1, 1:1.3 and 1:1.5, respectively (size distribution histograms can be found in Supporting Information File 5, Figure S6).

For all the decorated samples we have used the same source of acidic-functionalized MWCNTs. Accordingly, all the MWCNTs used have, more or less, the same defect size and distribution on the side walls. As the amount of iron salt increases, more iron precursor will be able to reach and interact with a larger number of defects on the MWCNTs side walls. Therefore, the density of the formed iron nanoparticles will increase. However, the average particle size of those nanoparticles will be the same because the side defects have the same size distribution for all samples.

**Figure 4:**
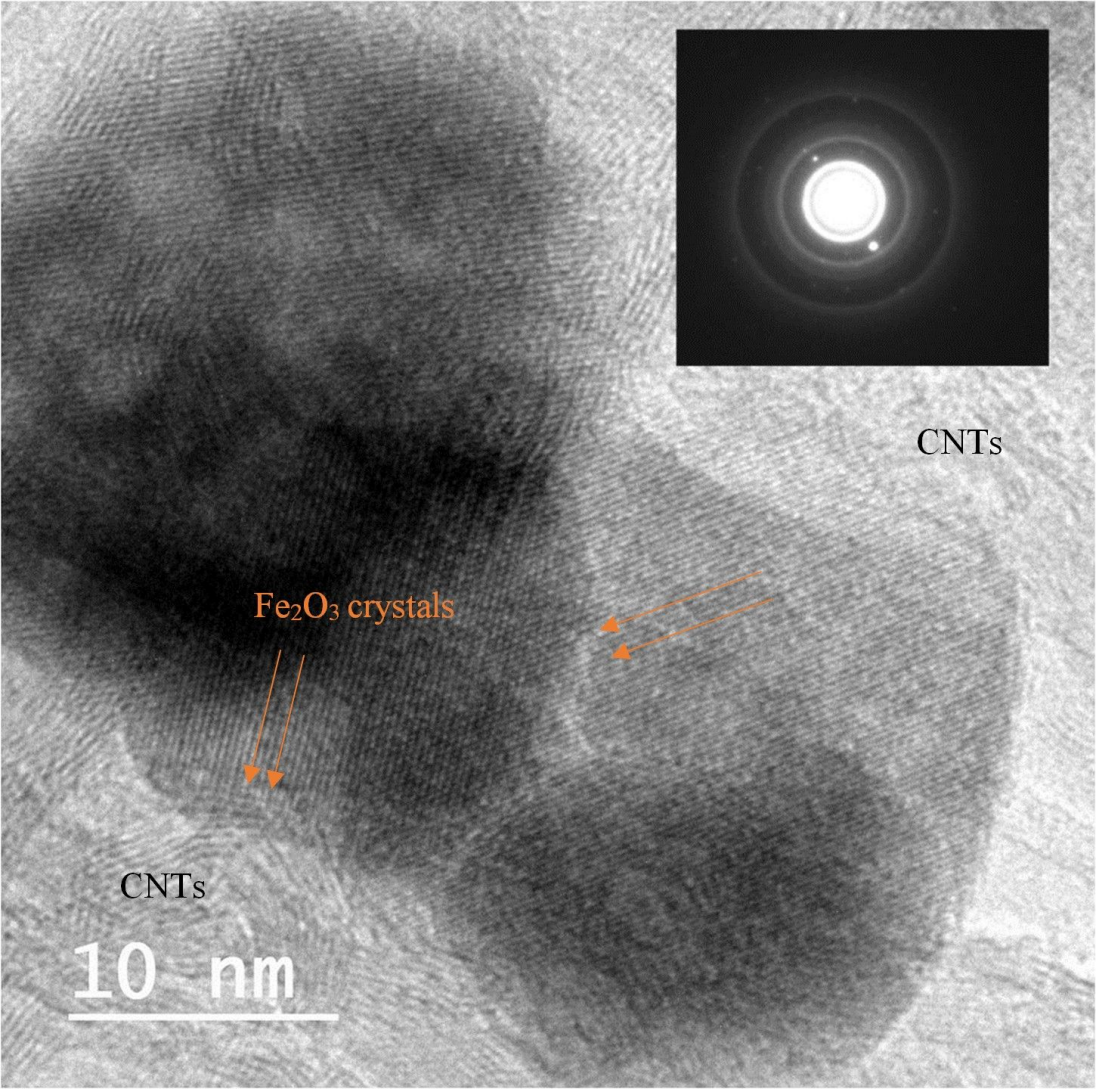
High magnification HRTEM images of MWCNTs decorated with Fe_2_O_3_ nanoparticles. The inset shows the electron diffraction pattern (SAED) for the selected area.

In addition, HRTEM imaging for the anchored iron oxide nanoparticles on the MWCNTs surface was performed and the selected area electron diffraction (SAED) pattern for was identified, as shown in Figure 4. The image shows the high crystallinity of the prepared iron oxide nanoparticles and the selected area electron diffraction (SAED) pattern of the iron oxide nanoparticles (see inset of Figure 4) clearly shows the diffraction rings of a typical cubic structure.

**Figure 5:**
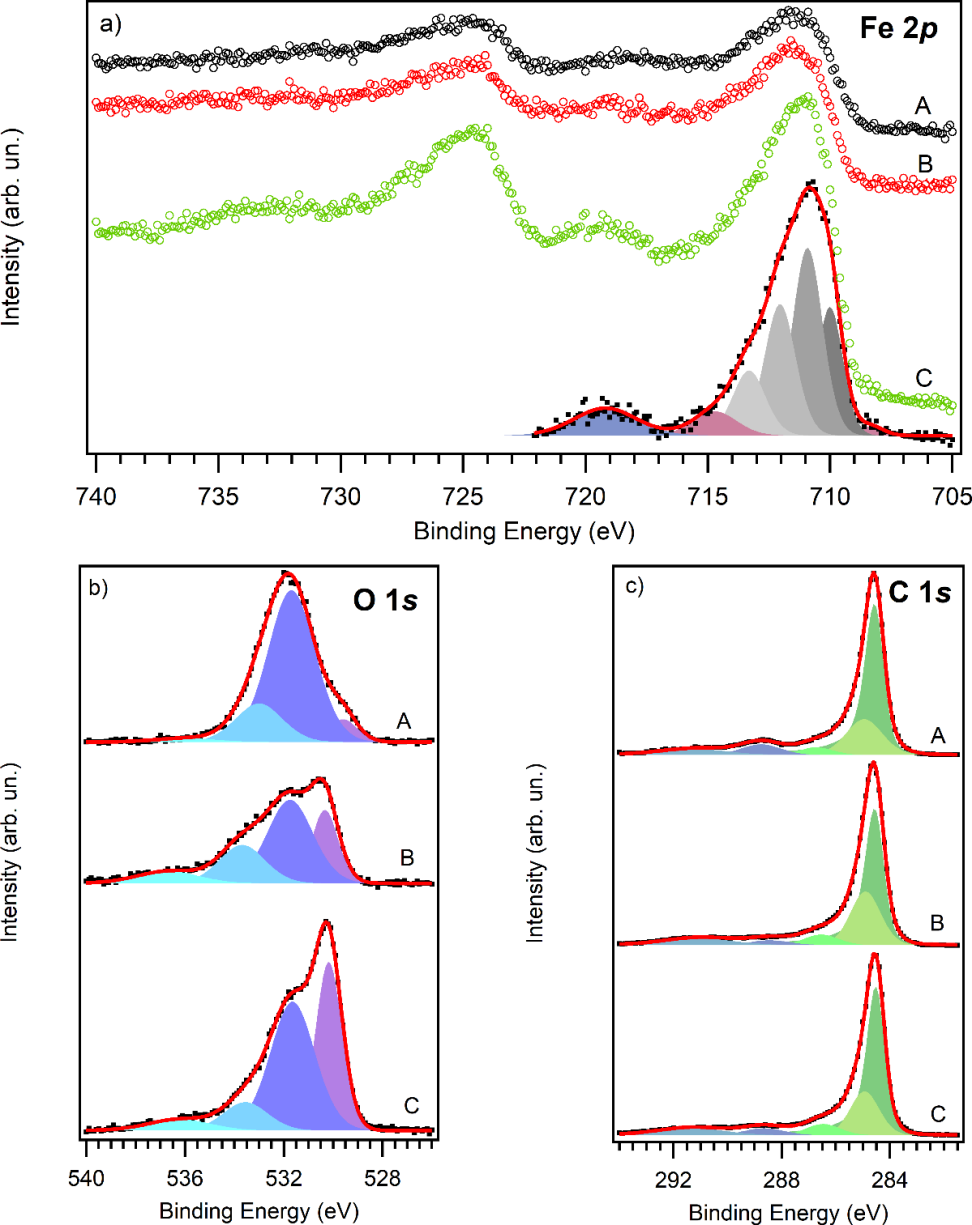
XPS core level spectra of Fe 2p with a fitting curve for sample C (a), O 1s (b) and C 1s (c) for the samples A (black curve), B (red curve) and C (green curve). The C 1s spectra has been normalized and aligned.

XPS was performed to investigate the chemical composition of the samples and, in particular, to determine the oxidation state of iron in the nanoparticles that decorate the CNT sidewalls. These results are shown in Figure 5. A description and coding of the samples analyzed as well as their chemical composition derived from the XPS analysis is summarized in Table 1. The values of the content for each element have been evaluated at different points on the sample and averaged, with an error as low as ±0.5%. The concentration of iron well reflects the decorating ratio, with sample C (1 CNT/1.5 Fe Salt) being the one with the highest Fe content. Residual nitrogen and sodium can be found in samples A and C respectively, which is probably due to some contamination during the fabrication process that we assume will not affect sensor performance.

**Table 1:** Description of the analyzed samples with the percent of different elements in each sample.

Sample	Description	C (%)	O (%)	Fe (%)

A	2nd decorating approach with a ratio of (1 CNT/1 Fe salt); not calcined	78.0	17.0	2.9
B	2nd decorating approach with a ratio of (1 CNT/1 Fe salt); calcined for 30 minutes	86.0	11.0	2.5
C	2nd decorating approach with a ratio of (1 CNT/1.5 Fe salt); calcined for 30 minutes	75.0	19.0	5.7

Figure 5a shows the typical Fe 2p XPS spectrum recorded on the studied samples. The spectrum is composed of two main structures centered at 712 and 725 eV corresponding to photoelectrons emitted from Fe 2p_3/2_ and Fe 2p_1/2_, respectively. Two satellite structures are also present centered at 719 and 733 eV. As can be seen in the figure, the Fe 2p_3/2_ region for sample C can be reproduced using a decomposition of four peaks (grey components), plus a surface peak (purple component) and a shake-up satellite (blue component) according to Grosvenor et al. [29]. The energy position of these peaks, in particular the first one of the 4-component multiplet (710.0 eV) and the satellite (719.2 eV), indicates the presence of iron in the Fe(III) oxidation state (Fe^3+^), characteristic of Fe_2_O_3_ and oxide-hydroxide. It is reported that the typical value for the satellite peak of Fe^2+^ (FeO) is 715.5 eV [30] and main 2p peak centered at 708 eV [31], while metallic iron has the main peak at much lower binding energy (706.7 eV).

The O 1s core level spectra, shown in Figure 5b, was reproduced using four peaks. The first one at 530.2 eV is attributed to oxygen in iron oxide: its contribution is higher in the spectra recorded on sample C, where the relative amount of iron was found to be the highest. The peak at 531.7 eV is mostly due to hydroxyl OH and O–C groups, while the remaining two peaks are attributed to other O–C groups and adsorbed water [32–33].

C–O contributions can be also observed in the C 1s core level spectra in Figure 5c by the presence of the peak at 288.6 eV. This contribution is higher in the spectrum recorded on sample A, where the highest amount of oxygen was found. The line shape of the C1s spectra recorded is typical for carbon nanotubes, with an asymmetric and narrow sp^2^ peak at 284.5 eV; this is followed by a second contribution due to carbon in amorphous or sp^3^ configuration at 285.0 eV [34]. The presence of these peaks associated with C–O bonds indicates that the functionalization of the CNTs (with COOH) is still present after the decoration process.

**Figure 6:**
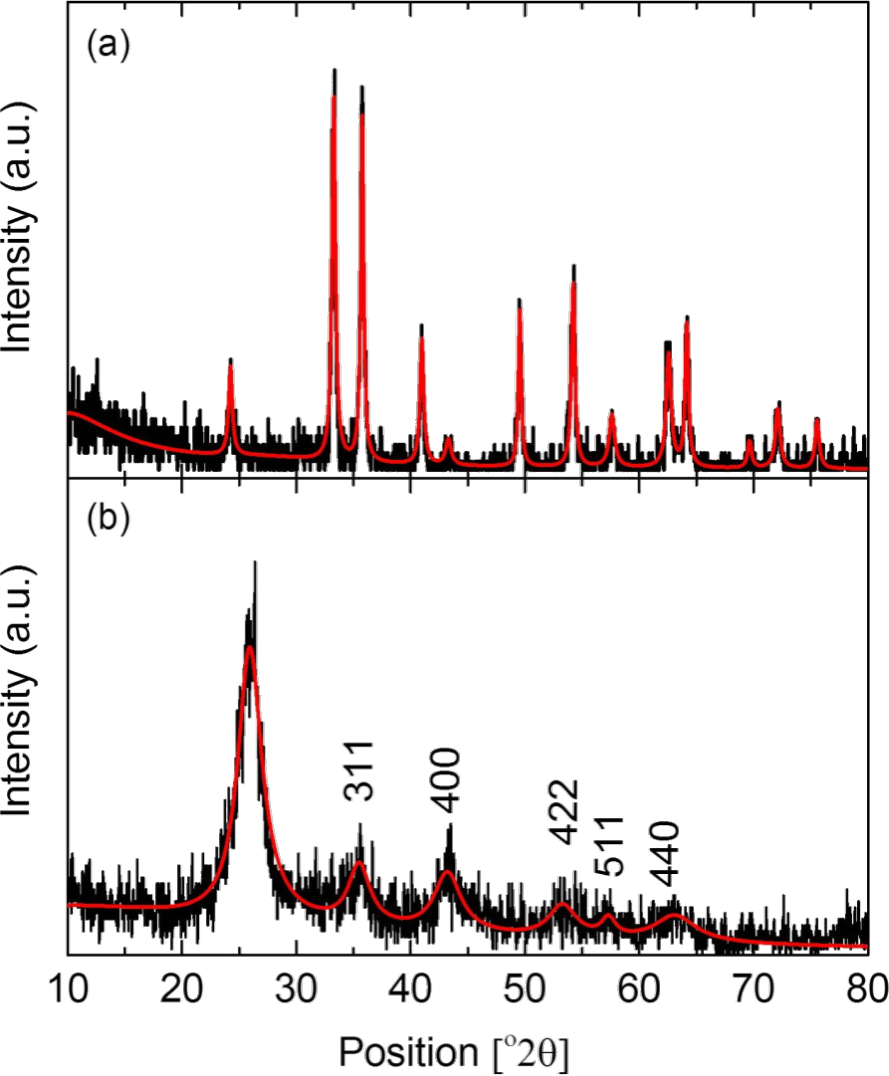
XRD pattern for Fe_2_O_3_ nanoparticles (a) and decorated CNTs with Fe_2_O_3_ nanoparticles (b).

The fact that the nanoparticles consisted of Fe_2_O_3_ was further confirmed by XRD characterization. For this purpose, pure iron oxide nanoparticles were prepared following the procedure described above. Figure 6 shows the spectra of the iron oxide nanoparticles and iron oxide nanoparticle-decorated nanotubes. These last results correspond to the sample with a 1:1 decoration ratio and calcined for 30 min. As it can be seen, the pattern of the Fe_2_O_3_ nanoparticles corresponds to a cubic crystalline structure, which confirms the HRTEM results. In the XRD pattern for Fe_2_O_3_/CNTs the characteristic peak at 25.994° attributed to plane (002) of the CNTs can be clearly identified. The other diffraction peaks at 35.6°, 43.15°, 53.28°, 57.3°, 63.12° can be attributed to planes (311), (400), (422), (511) and (440) of the cubic Fe_2_O_3_ phase.

### Gas sensing properties

**Figure 7:**
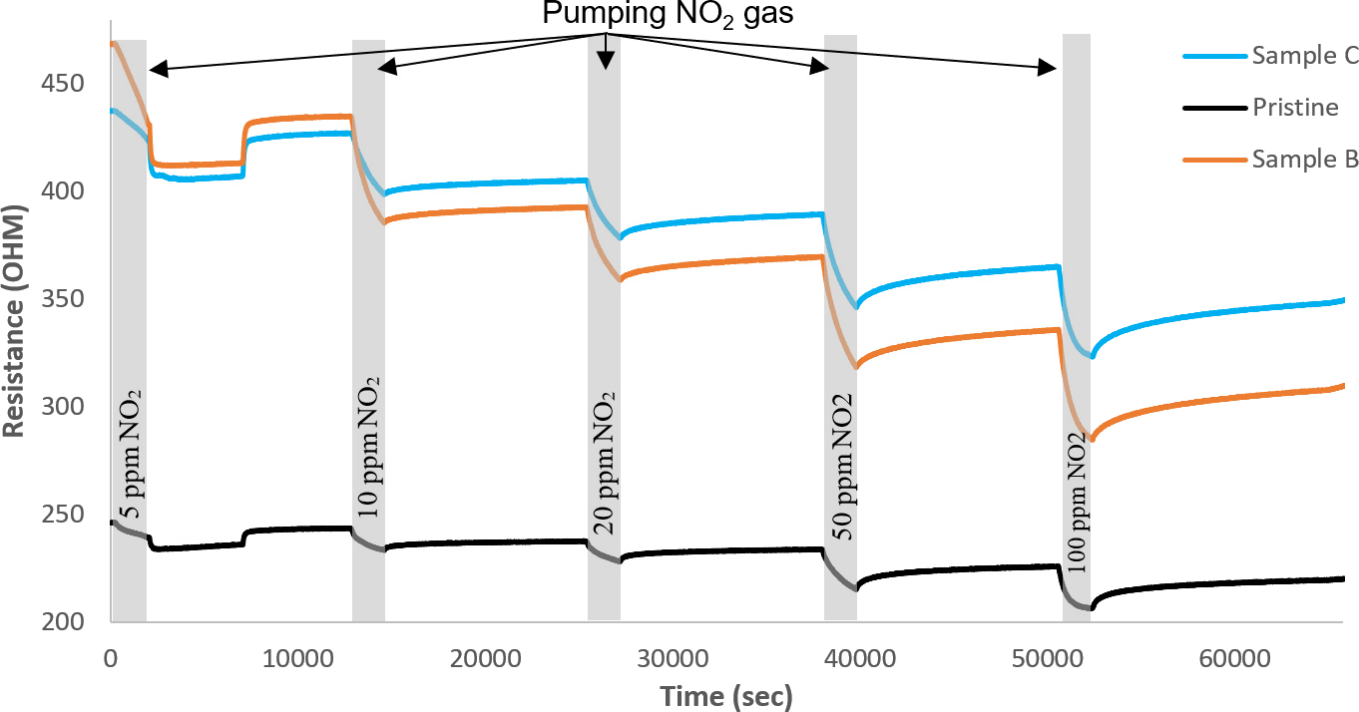
Electrical resistance of the samples as a function of time.

Samples B and C were used to prepare sensors using the drop-coating approach to check the effect of decoration ratio on the response. An additional sensor was prepared using pristine carbon nanotubes with the airbrushing approach. Figure 7 shows electrical resistance against time.

Nitrogen dioxide was found to strongly interact with carbon nanotube sensors, and as a result, the sensors did not fully recover their baseline resistance value during the cleaning phase, which was conducted at room temperature without heating. Applying mild heating or UV light have been reported useful for fully recovering the baseline after exposure to nitrogen dioxide [10]. Therefore, for calculating the response to any given nitrogen dioxide concentration, the value of *R*_0_ was taken as the value of the sensor resistance before being exposed to the corresponding gas concentration and the value of *R*_G_ was fixed as the value of resistance at a fixed time after an NO_2_ exposure of 10 minutes.

**Figure 8:**
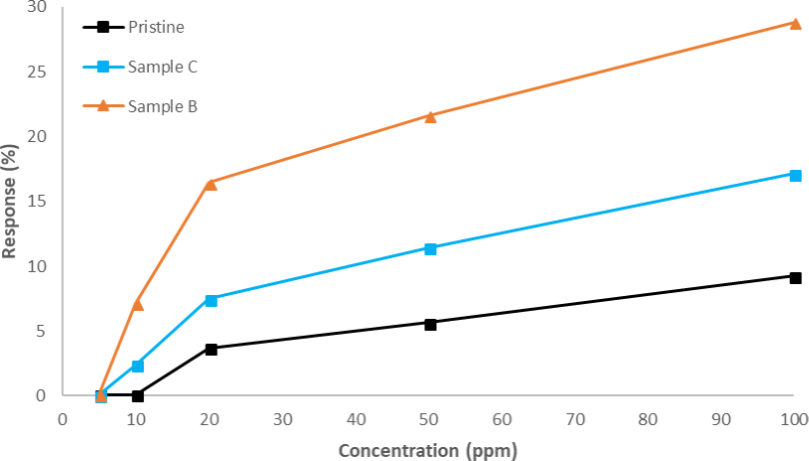
Effect of decoration ratio on the gas sensing performance.

Figure 8 shows the calibration curves for different concentrations of NO_2_. Response (%) is defined as 100 × (*R*_G_ – *R*_0_)/*R*_0_. As derived from Figure 8, Sample B with a 1:1 decoration ratio shows better response than sample C and obviously better than pristine CNTs.

These results allow us to conclude that the decoration with iron oxide improves sensor performance in the detection of NO_2_. Regarding the amount of iron oxide introduced, the best result is obtained for the lower decoration ratio of 1:1 which is considered to be the optimum decoration ratio.

By comparing these results to other results in the literature, we can conclude that there is an optimum decoration ratio which gives us the highest response, as the response increases with increasing decoration ratio dose until an optimum decoration density is reached and afterwards the response decreases [6,19,28]. In fact, the obtained results are better regarding the intensity of the sensor response, as compared to those obtained by Chuanfei Hua et al. [23] using a composite of SWCNT–Fe_2_O_3_, although their sensors show faster response time.

### Studying effect of calcination period on nanocluster size

**Figure 9:**
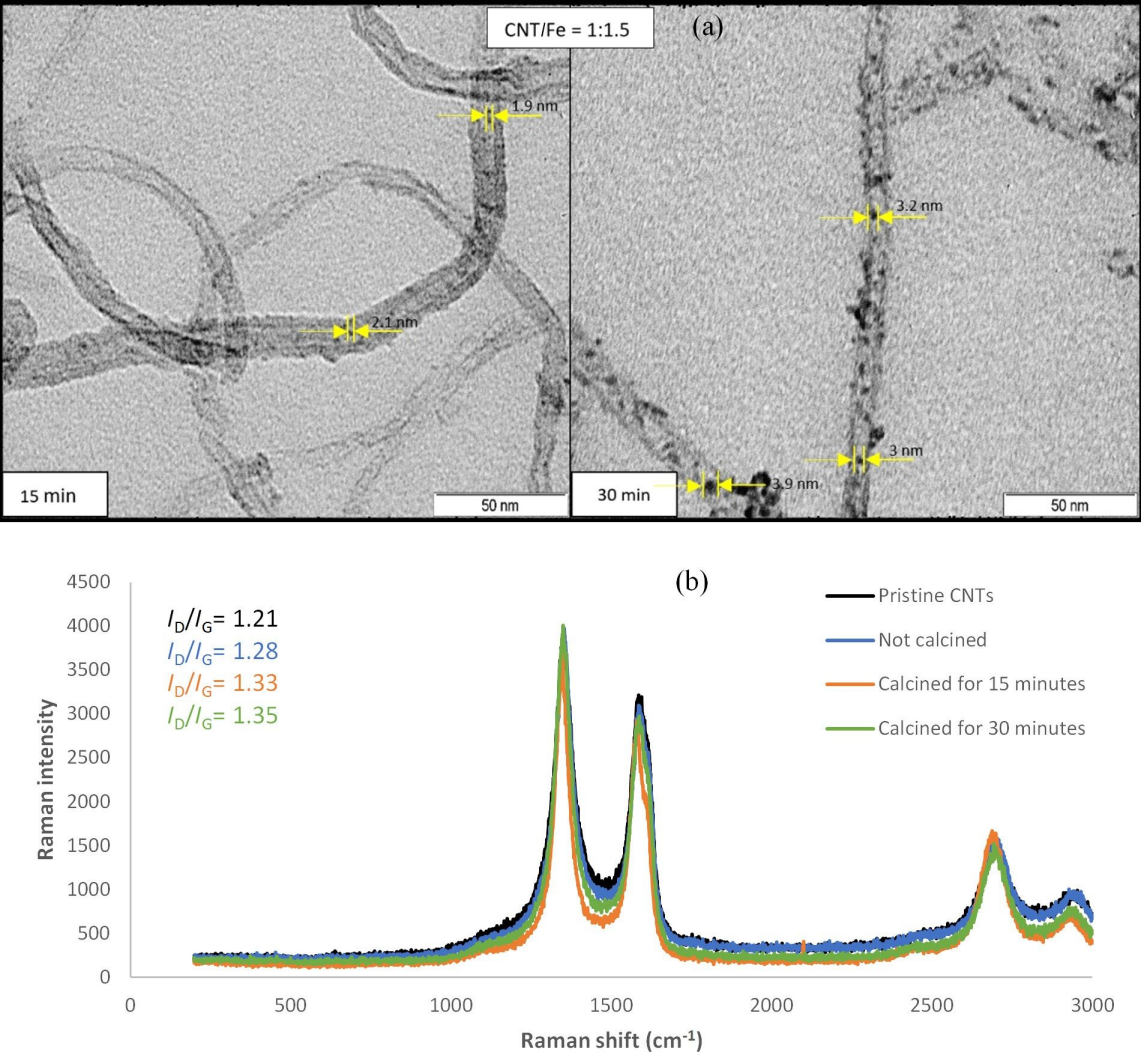
TEM images showing nanocluster size (a) after calcination for 15 minutes and 30 minutes for a 1:1.5 decoration ratio and Raman spectra for pristine CNTs and decorated CNTs with different calcination periods for (1:1.5) decorated COOH–CNTs (b).

TEM images for two samples with the same decoration ratio (CNT/Fe = 1:1.5) but with different calcination periods of 15 or 30 minutes were taken to investigate the effect of the duration of the calcination on the size of iron nanoclusters. This is shown in Figure 9a. In addition, the nanocluster size distribution can be found in Supporting Information File 5, Figure S5. It can be concluded that the nanocluster size increases with increasing calcination time.

Also, the Raman spectra for pristine CNTs along with decorated samples of CNT/Fe = 1:1.5, which were calcined for 15 or 30 minutes, were studied to determine the effects of the calcination period on the quality of CNTs in comparison to non-calcined decorated CNTs. These results are shown in Figure 9b.

By analyzing the Raman spectra, we conclude that by increasing the calcination time, the quality of the CNTs slightly decreases. We also notice that the change in *I*_D_/*I*_G_ between pristine and decorated CNTs is not very high. This is expected because the commercial CNTs provided from Nanocyl are of low purity (95%) in analytical terms. In addition, the CNTs were already functionalized, so it is logical that a relatively high D/G ratio is obtained before performing any treatment or decoration. This low crystallinity means that a high concentration of disordered sp^2^ carbon in relation to the presence of stretching C–C bonds is already present in as-purchased CNT samples. This makes it difficult to significantly increase defects in CNTs after performing further treatment and decoration.

**Figure 10:**
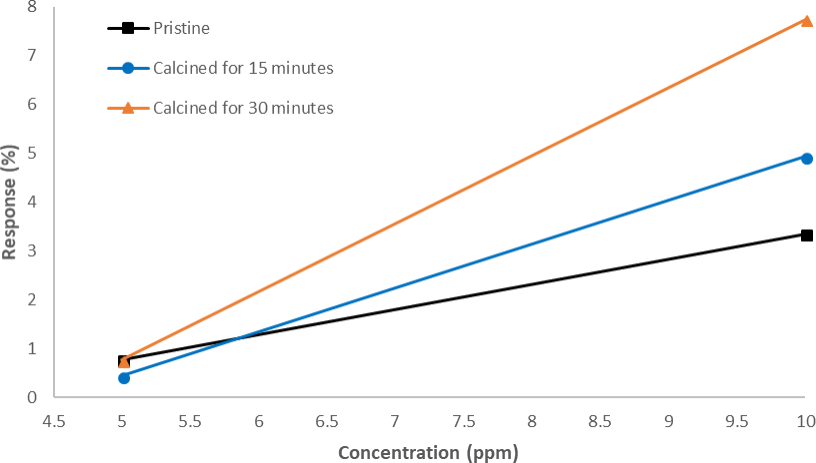
Effect of calcination period on the gas sensing performance.

In order to check the influence of nanoparticle size on the sensing capabilities of the CNTs, carbon nanotubes corresponding to sample A calcined for 15 or 30 minutes were used to implement sensors by the drop-coating approach. The previous sensor based on pristine CNTs was also used for comparison. Two different concentrations of NO_2_ gas (5 ppm, 10 ppm) were pumped into the test chamber in this case. The results are shown in Figure 10.

As shown in Figure 10, the response of the sensor that employed carbon nanotubes calcined for 30 minutes, i.e., the one with larger iron oxide particles, is higher than the sensor based on iron oxide decorated CNTs calcined for 15 minutes.

These results confirm again that the decoration with iron oxide enhances the sensor response to NO_2_, obtaining better results for carbon nanotubes decorated with iron oxide nanoparticles of larger size. This is consistent with the literature, in which heat treatment on sensors doped with an optimum doping ratio can cause both an increase in the size of decorating nanoparticles and an enhancement in the response of the sensor [6].

In order to check if the deposition method (drop-coating thick film or airbrushing thin film) has an influence on the sensor behavior, an additional sensor using CNTs corresponding to sample B was prepared by air brushing. Differences in morphologies between the two approaches followed for the deposition procedure (drop coating and air brushing) can be found in Supporting Information File 1, Figure S1.

This new sensor, together with the one based on pristine nanotubes fabricated using the same approach, and the sensor based on CNTs of sample B, but obtained by drop coating were tested.

**Figure 11:**
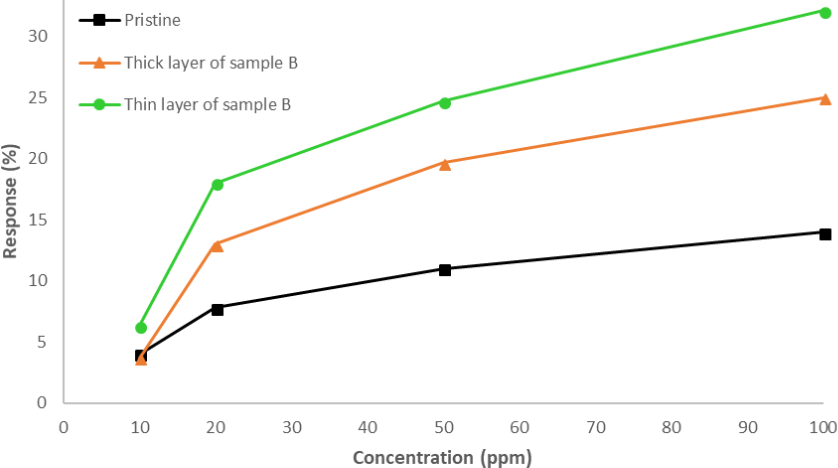
Effect of layer homogeneity and thickness on the gas sensing performance.

The results are shown in Figure 11. As it can be seen, the thin layer sensor obtained by airbrushing decorated nanotubes showed better response than the thick film sensor obtained by drop coating. Once more, the results confirm that the decoration of the nanotubes using iron oxide is a good approach to enhance the sensor response to NO_2_. In this case, we are comparing sensors implemented employing the same procedure, using both decorated and pristine nanotubes. Moreover, the airbrushed sensor showed the best response.

In order to check the selectivity of the best performing sensor, measurements for 10 ppm of benzene and 100 ppm of CO were performed. Although the concentrations of both gases were quite high, the sensor showed no response to carbon monoxide while the response to benzene was lower than 0.06%, confirming a good selectivity for the target gas (i.e., nitrogen dioxide).

**Figure 12:**
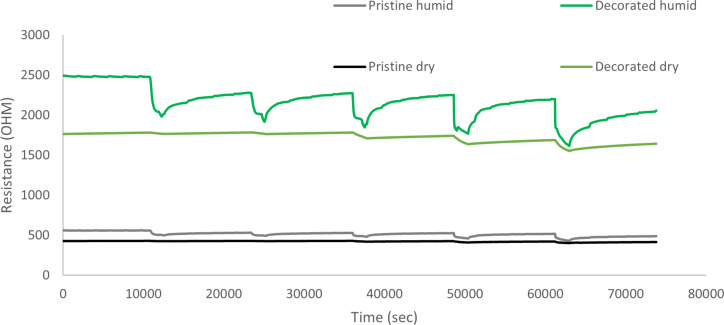
Comparison between gas sensors – performance in both dry and humid conditions.

Finally, to check the effect of humidity in the performance of the sensor, a new set of measurements for NO_2_ were performed. In this case, the relative humidity was set to 50%. Comparing these results with the ones performed with dry air (relative humidity was around 3%), one can realize that the sensor, when working in a more humid environment, shows faster response. That is, the presence of water vapor improves the performance of the sensor. The results of these measurements are shown in Figure 12. This enhancement in nitrogen dioxide response under humid conditions can be attributed to the water mediated adsorption of NO_2_ on iron oxide nanoparticles, as previously reported for semiconductor metal oxide chemoresistors [35].

A deeper analysis of the sensor behavior reflected in Figure 7 and Figure 11 shows that both pristine and decorated CNT films behave as a p-type semiconductor. When nitrogen dioxide reacts with the active layer, the molecule traps electrons from the active layer, increasing its conductivity, because this increases the concentration of holes, which act as main charge carriers.

Comparing the behavior of pristine and decorated CNTs, one can clearly see that the decoration process leads to an increase in the electrical resistance of the active layer. This fact can be attributed to the p–n junctions formed between the p-type CNTs and the n-type iron oxide nanoparticles, with the formation of associated depletion layers. The p-type behavior of the decorated nanotubes suggests that, when the sensor is exposed to NO_2_, the response is mainly due to the CNTs. In this case, the iron oxide NPs contribute to the enhancement of the response via a reduction of their associated depletion layer when NO_2_ molecules adsorb on the surface of NPs, which increases the conductivity of the layer. Nevertheless, there is another possible explanation. It has been reported that Fe_2_O_3_ can turn from n-type to p-type, especially in oxidizing ambient environments [36]. This possible change in the semiconducting behavior of Fe_2_O_3_ could be the reason why the response to other reducing gases such as benzene or CO has been found to be very low. Nevertheless, a deeper study is necessary to better determine the mechanisms responsible for sensor response.

## Conclusion

The decoration of MWCNTs with Fe_2_O_3_ using an inexpensive method based on wet chemistry has shown to be a good approach for enhancing the detection of NO_2_. The presence of iron oxide has been confirmed by both XPS and XRD analysis. Parametric studies for the decoration procedure showed that the decoration density is proportional to the ratio of CNT/Fe salt, without affecting particle size. The solvents used in the decoration steps affect the decorated CNT’s morphology, decoration uniformity and decoration homogeneity. Methanol and ethanol were found to allow for a more uniform and homogenous decoration along the CNTs and also better powder morphology. On the other hand, DMF and acetone resulted in the formation of agglomeration islands on the CNTs and negatively affected the uniformity and homogeneity of decoration. The effect of the calcination period on the size of the decorating nanoclusters was studied as well. It was found that their size increases by increasing the calcination period. Regarding the gas sensing effect of the decoration, lower decoration density with higher particle size led to the best results.

The effect of the deposition method was also studied and was found to affect the behavior of the sensor. Namely, thinner, homogeneous, layered films obtained by airbrushing showed better response than thicker, non-homogeneous, layered sensors obtained by drop coating.

This last sensor deposited by air brushing showed an excellent selectivity for NO_2_ when carbon monoxide and benzene vapors were considered as potential interfering gases. Finally, the effect of humidity was studied. It was found that a more humid environment resulted in an increased and faster response of the sensor to NO_2_. This effect was observed for both pristine and decorated sensors.

## Supporting Information

The supporting information features images of the effect of deposition technique on decorated carbon nanotubes, images of the wire bound sensor (both sides), images of the teflon gas sensing chamber, an image of the results from the first decoration method on carbon nanotubes, and a results showing the effect of the calcination period of the nanoparticle size.

File 1 Effect of deposition technique on decorated carbon nanotube morphology.

File 2 Wire bonded sensor.

File 3 Teflon gas sensing chamber allowing for 4 different sensors together for gas sensing.

File 4 Effect of first decoration step on carbon nanotube morphology.

File 5 Nanoparticle size distribution histograms.
