# Supplementary material for: Wet chemistry route for the decoration of carbon nanotubes with iron oxide nanoparticles for gas sensing
Source: Beilstein J Nanotechnol. 2019 Jan 9;10:105–18. doi: 10.3762/bjnano.10.10 (PMC6334796; doi:10.3762/bjnano.10.10)
Supplement: File 1 — Effect of deposition technique on decorated carbon nanotube morphology. [file Beilstein_J_Nanotechnol-10-105-s001.pdf]

BEILSTEIN JOURNAL OF NANOTECHNOLOGY

Supporting Information

for

Wet chemistry route for the decoration of carbon nanotubes with iron oxide nanoparticles for gas sensing

Hussam M. Elnabawy, Juan Casanova-Chafer, Badawi Anis, Mostafa Fedawy, Mattia Scardamaglia, Carla Bittencourt, Ahmed S. G. Khalil, Eduard Llobet and Xavier Vilanova

Beilstein J. Nanotechnol. **2019,** *10,* 105–118. doi:10.3762/bjnano.10.10

Effect of deposition technique on decorated carbon nanotube morphology

License and Terms: This is a supporting information file under the terms of the Creative Commons Attribution License (http://creativecommons.org/licenses/by/4.0). Please note that the reuse, redistribution and reproduction in particular requires that the authors and source are credited.
The license is subject to the *Beilstein Journal of Nanotechnology* terms and conditions: (https://www.beilstein-journals.org/bjnano)

As mentioned in the experimental part of the work, there were two methods for deposition of CNTs layer. The first approach, using drop by drop technique, lead to a non-homogeneous thick layer, while the second approach, using air-brushing technique, lead to a more homogeneous thin layer. The different morphologies obtained for both approaches can be seen in Figure S1.

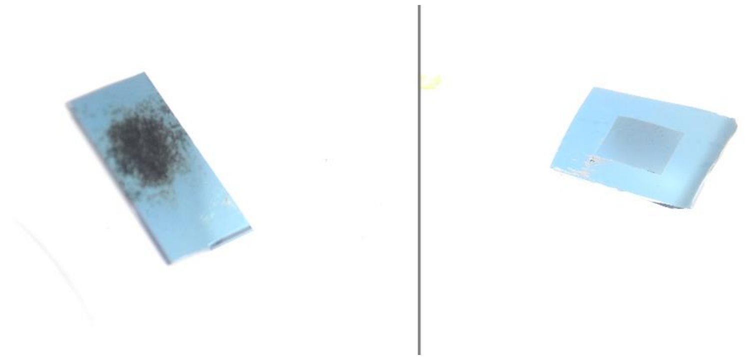

Figure S1: CNTs layer deposited with different morphology; thick layer resulting from first approach (a) and thin layer resulting from second approach (b).
