# Supplementary material for: Wet chemistry route for the decoration of carbon nanotubes with iron oxide nanoparticles for gas sensing
Source: Beilstein J Nanotechnol. 2019 Jan 9;10:105–18. doi: 10.3762/bjnano.10.10 (PMC6334796; doi:10.3762/bjnano.10.10)
Supplement: File 2 — Wire bonded sensor. [file Beilstein_J_Nanotechnol-10-105-s002.pdf]

BEILSTEIN JOURNAL OF NANOTECHNOLOGY

Supporting Information

for

Wet chemistry route for the decoration of carbon nanotubes with iron oxide nanoparticles for gas sensing

Hussam M. Elnabawy, Juan Casanova-Chafer, Badawi Anis, Mostafa Fedawy, Mattia Scardamaglia, Carla Bittencourt, Ahmed S. G. Khalil, Eduard Llobet and Xavier Vilanova

Beilstein J. Nanotechnol. **2019,** *10,* 105–118. doi:10.3762/bjnano.10.10

Wire bonded sensor

License and Terms: This is a supporting information file under the terms of the Creative Commons Attribution License (http://creativecommons.org/licenses/by/4.0). Please note that the reuse, redistribution and reproduction in particular requires that the authors and source are credited.
The license is subject to the *Beilstein Journal of Nanotechnology* terms and conditions: (https://www.beilstein-journals.org/bjnano)

As mentioned in experimental part of the work, sensors were fabricated by depositing a layer of decorated carbon nanotubes on a small rectangular piece of oxidized silicon, heaters were attached to the back side of the silicon substrate then connections between BCP and both heater and active layer decorated carbon nanotubes were made using platinum wires. Both top and bottom views for the sensors are shown in Figure S2.

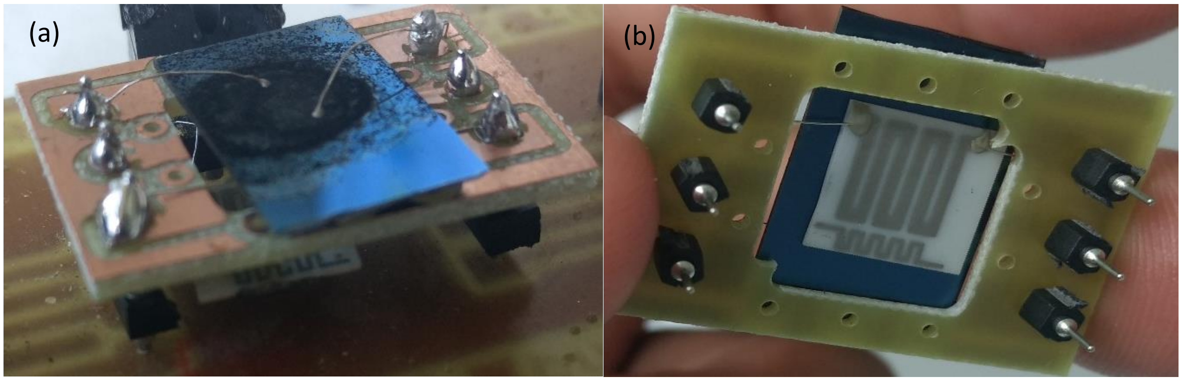

Figure S2: (a) Top and (b) bottom views of the fabricated sensor.
