# Supplementary material for: Wet chemistry route for the decoration of carbon nanotubes with iron oxide nanoparticles for gas sensing
Source: Beilstein J Nanotechnol. 2019 Jan 9;10:105–18. doi: 10.3762/bjnano.10.10 (PMC6334796; doi:10.3762/bjnano.10.10)
Supplement: File 5 — Nanoparticle size distribution histograms. [file Beilstein_J_Nanotechnol-10-105-s005.pdf]

BEILSTEIN JOURNAL OF NANOTECHNOLOGY

Supporting Information

for

Wet chemistry route for the decoration of carbon nanotubes with iron oxide nanoparticles for gas sensing

Hussam M. Elnabawy, Juan Casanova-Chafer, Badawi Anis, Mostafa Fedawy, Mattia Scardamaglia, Carla Bittencourt, Ahmed S. G. Khalil, Eduard Llobet and Xavier Vilanova

Beilstein J. Nanotechnol. **2019,** *10,* 105–118. doi:10.3762/bjnano.10.10

Nanoparticle size distribution histograms

License and Terms: This is a supporting information file under the terms of the Creative Commons Attribution License (http://creativecommons.org/licenses/by/4.0). Please note that the reuse, redistribution and reproduction in particular requires that the authors and source are credited.
The license is subject to the *Beilstein Journal of Nanotechnology* terms and conditions: (https://www.beilstein-journals.org/bjnano)

1. Effect of calcination period on nanocluster size

Figure S5 shows a chart of the different size distribution for two samples of the same decoration ratio of (CNT/Fe = 1:1.5) but with different calcination periods as TEM images, shown in Figure 9a of experimental part from the work, were analyzed using ImageJ software and sizes of different number of particles were measured then the average size was calculated.

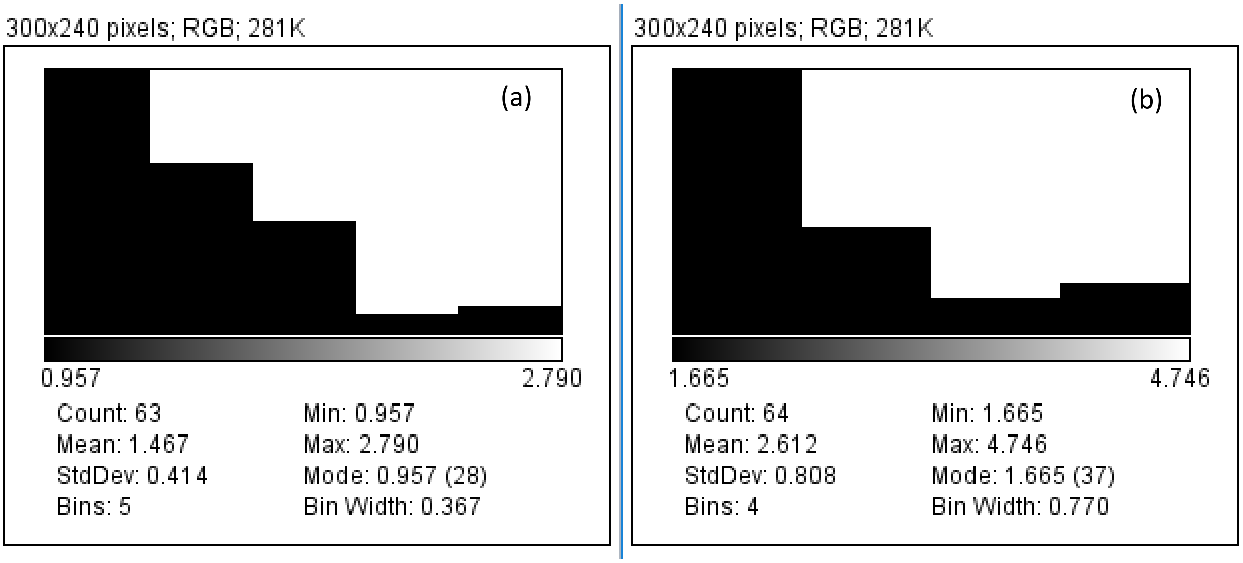

Figure S5: Nanocluster size distribution in case of 15 minutes calcination (a) and 30 minutes calcination (b).

2. Effect of decoration ratio on nanocluster size

Figure S6 shows a chart of the size distribution for three samples with the same calcination time (30 minutes) but for decoration ratios of (CNT/Fe = 1:1, 1:1.3 and 1:1.5). As TEM images, shown in Figure 3 of experimental part from the work, were analyzed using ImageJ software and sizes of different number of particles were measured then the average size was calculated to determine the effect of decoration ratio on the nanocluster size.

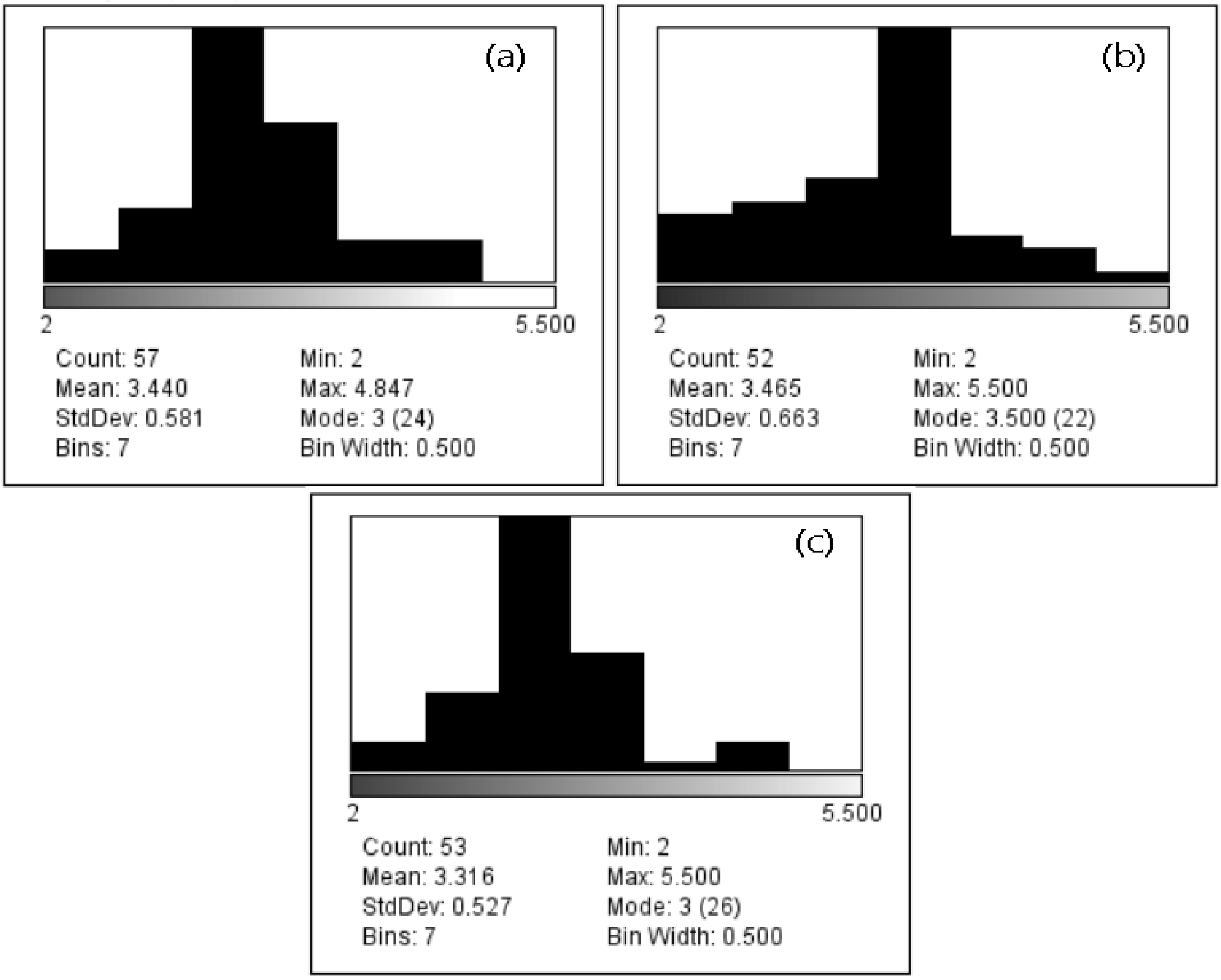

Figure S6: Nanocluster size distribution in case of 30 minutes calcination for decoration ratio of 1:1(a), decoration ratio of 1:1.3(b) and decoration ratio of 1:1.5 (c).
